# Vitamin D: the tricky hormone

**DOI:** 10.1590/2359-3997000000250

**Published:** 2017-01-01

**Authors:** Victória Zeghbi Cochenski Borba

**Affiliations:** 1 Department of Internal Medicine Universidade Federal do Paraná Brasil Department of Internal Medicine, Universidade Federal do Paraná (UFPR); Serviço de Endocrinologia e Metabologia do Hospital de Clínicas da Universidade Federal do Paraná (SEMPR)

In this issue of *The Archives of Endocrinology and Metabolism* two papers investigated the impact of vitamin D in two different health conditions: one showed a negative result investigating the association of vitamin D levels with infertility and the other one the positive effect of vitamin D supplementation in the physiological lung function.

Vitamin D, a pre-hormone produced in the skin under sunlight exposure, undergoes various metabolic steps until its active form, 1,25-dihydroxyvitamin D3, is formed. The intermediary form, 25-hydroxyvitamin D (25OHD), is the recommended form for monitoring vitamin D status. The presence of 1α-hydroxylase (CYP27B1) and the vitamin D receptor (VDR) in many cells indicates a possible local ability to synthesize 1,25-dihydroxyvitamin D3, the active metabolite of vitamin D, which has turned the attention of the medical community to the noncalcemic effects of vitamin D (1). In recent decades, several studies have demonstrated the association of circulating 25OHD levels with different diseases, their activity, and health conditions. A positive association was found in several disorders such as systemic lupus erythematosus (SLE) and its activity (2); chronic obstructive pulmonary disease (COPD) (3); inflammatory bowel disease (IBD) (4); systemic sclerosis; endocrine disorders; cancer; immune; cardiovascular and other chronic diseases (1,5); despite the presence of numerous positive observational studies, causation studies are lacking. A few good-quality randomized controlled studies on vitamin D supplementation in diverse disease endpoints show a modest effect on blood pressure, glycemic control, cancer, and immune response (5,6).

Hypovitaminosis D is highly frequent worldwide and in Brazil, the prevalence ranges from 16% to 80% depending on the latitude and the population studied (1,7). Although low levels of vitamin D are present in different diseases, infertile women studied in the article by Lopes and cols. (8) and also by others (9) did not show lower levels compared to controls. The literature in the field is ambiguous, showing both the importance of its adequate level and the lack of effect in infertility issues, such as polycystic ovary syndrome, endometriosis, myoma-induced infertility, male infertility, premature ovary failure and *in vitro* fertilization techniques (10,11). Interestingly, however, Lopes and cols. (8) pointed out the high prevalence of hypovitaminosis D in a young premenopausal population, which is in agreement with the low vitamin D levels described for postmenopausal and older women in Brazil. Vitamin D and its level of impact on fertility is a matter to be defined. This controversy points out the wide effect of vitamin D and the need for a better understanding of its metabolism.

Nolasco and cols. (12) showed a positive effect of vitamin D on pulmonary function in healthy postmenopausal women participating in an aquatic exercise program. Low vitamin D levels were previously described in pulmonary diseases as well as their impact in the beginning and disease progression (13). However, studies examining the effect of vitamin supplementation on pulmonary diseases have shown diverse results. The response to tuberculosis treatment with and without vitamin D was not different (14,15). Even though low vitamin D levels was associated with impaired lung function and to an inadequate response to treatment in patients with asthma, the addition of vitamin D did not modify the progression of the disease, compared to controls (16,17). The study published in this issue, showing improvement in pulmonary function with vitamin D, confirms once again the controversy around vitamin D.

The controversy could be explained by diverse factors such as the lack of assessment of long-term, overall vitamin D intake as a dietary source, or a long-term evaluation of vitamin D levels; the sometimes unavailable measurement of inactive and active metabolites; the complexity of vitamin D metabolism and the variation of the methodology of measurement; and the high inter-individual variability in vitamin distribution between carrier proteins and target receptors (18). Many trials are ongoing in an attempt to clarify the impact of vitamin D in different physiological or medical aspects. Whether or not they succeed is a matter of debate due to the variability of protocols.

Considering all these aspects, we can give Vitamin D the title “The Tricky Hormone”, as the medical community is challenged to clarify the real impact of this complex hormone.
